# Probiotics: Potential Novel Therapeutics Against Fungal Infections

**DOI:** 10.3389/fcimb.2021.793419

**Published:** 2022-01-21

**Authors:** Yunjian Wu, Shan Hu, Changyu Wu, Feng Gu, Ying Yang

**Affiliations:** ^1^ Department of Biotechnology, Beijing Key Laboratory of New Molecular Diagnosis Technologies for Infectious Diseases, Beijing Institute of Radiation Medicine, Beijing, China; ^2^ School of Medical Imaging, Xuzhou Medical University, Xuzhou, China; ^3^ Department of Laboratory Medicine, Xuzhou Tumor Hospital, Xuzhou, China; ^4^ Department of Laboratory Medicine, Xuzhou Central Hospital, Xuzhou, China

**Keywords:** probiotics, fungal infections, antifungal, biofilms, therapeutic intervention

## Abstract

The global infection rate of fungal diseases is increasing year by year, and it has gradually become one of the most serious infectious diseases threatening human health. However, the side effects of antifungal drugs and the fungal resistance to these drugs are gradually increasing. Therefore, the development of new broad-spectrum, safe, and economical alternatives to antibacterial drugs are essential. Probiotics are microorganisms that are beneficial for human health. They boost human immunity, resist pathogen colonization, and reduce pathogen infection. Many investigations have shown their inhibitory activity on a wide range of pathogenic fungi. However, their antibacterial mechanism is still a secret. This article reviews the progress of probiotics as a new method for the treatment of fungal diseases.

## Introduction

Fungal infections are classified as superficial fungal infections or deep fungal infections according to the location of the disease occurrence. Superficial fungal infections are common on the skin and nails, such as hand tinea, tinea pedis, and tinea unguium. They are often highly contagious, but not life-threatening. Deep fungal infection threatens human life, and the death rate of infected patients is extremely high (Dellière et al., 2020; Vitális et al., 2020). Every year, more than 1.5 million people die worldwide, as a result of deep fungal infections. Statistical reports show that even if patients receive antifungal treatment, the mortality rate after illness is more than 50% (Brown et al., 2012). Patients frequently experience missed and misdiagnosed circumstances due to the lack of particular clinical symptoms and signs associated with deep fungal infections, as well as the lack of rapid detection methods. Owing to this, the actual situation of deep fungal infection is more severe than reported (Hu et al., 2021a).

Currently, four types of antifungal drugs are commonly used in clinical practice, including azoles, polyenes, pyrimidines, and echinocandins (Oltu et al., 2020). These antifungal drugs have considerable side effects, with various limitations (Sant et al., 2016; Perlin, 2020). Pathogenic fungal resistance has become extremely serious, and the outbreak of multidrug-resistant Candida has caused widespread concern globally (Hu et al., 2021b). Therefore, it is essential to develop safe and more effective drug alternatives to treat deep fungal infections.

Probiotics are non-toxic and have no side effects. They have high stability and inhibit pathogenic bacteria (Ozen and Dinleyici, 2015; Suez et al., 2019). Probiotics have performed well in treating gastrointestinal diseases and are currently a beneficial antifungal application (Kim et al., 2019; Miles, 2020). Many scientists have recently discovered that probiotics can also inhibit the growth of fungi (Hu et al., 2017; Shenoy and Gottlieb, 2019). From this perspective, probiotics can be used as a substitute for antifungal drugs.

## Commonly Used Antifungal Drugs

The global spread of multidrug-resistant fungus poses a significant challenge to infectious disease prevention and control in medical facilities (Matthew et al., 2018). The incidence of fungal infections in patients is gradually increasing due to the aggravation of the aging problem in many countries and regions, as well as the widespread use of invasive treatments (Enoch et al., 2017). At the same time, while the survival rate of cancer patients and organ transplant patients is improving, these patients are becoming increasingly susceptible to secondary infections from opportunistic fungi, necessitating the use of antifungal drugs in clinical practice (Ramirez-Garcia et al., 2016; Aslam and Rotstein, 2019). The abuse of antibiotics and antifungal drugs can easily lead to the development of drug resistance in fungi. Long-term drug treatment not only invalidates the effect of the available antibacterial drugs but also leads to an imbalance of the human flora and a decline in the body’s immunity, making invasive fungal infections more plausible (Meng et al., 2020). If the situation continues in this way, a vicious cycle of antifungal drugs and drug-resistant fungi will emerge.

Systemic antifungal drugs have been the primary treatment for fungal infections since the first antifungal drug was introduced sixty years ago (Dutcher, 1968). While chemically synthesized medications can prevent fungi from growing, fungi can quickly develop resistance to them. This is due to the small size of the fungal genome in comparison to the human genome. Additionally, fungus proliferates faster, allowing it to quickly obtain variants that adapt to the new environment (Latgé and Chamilos, 2019).

Antifungal drug research progress has been very slow after 60 years of research. There are only four types of fungal therapeutic drugs that are currently used in clinical practice. The first category includes the most commonly used fungicides, azole drugs, which inhibit fungal growth by inhibiting ergosterol synthesis in fungal cell membranes. Common fungi are more resistant to these drugs, especially *Candida auris.* It has a resistance rate of about 80% to fluconazole (Howard et al., 2020; Hu et al., 2021b). Three hot-spot amino acid substitutions (Y132F, K143R, and F126L) have been identified after whole-genome sequencing of 47 C*. auris* clinical isolates (Lockhart, 2019). These mutations had previously been implicated in azole resistance in *C. albicans.* When the susceptible strain mutates and harbors these mutations, it develops drug resistance. Polyene drugs, such as amphotericin B, are the second class of antifungal drugs. These types of drugs have an antibacterial effect because they bind ergosterol to the lipid bilayer, destroying the cell membrane structure of the fungus. This class of drugs is mainly used against visceral or systemic infections caused by *Cryptococcus*, *Coccidioides*, *Histoplasma capsularis*, *Blastomyces*, *Sporothrix*, *Candida*, *Mucor*, *Aspergillus*, etc. The drug has a lot of toxicity and side effects, and there are a lot of cases of drug resistance (Matsumori et al., 2005). Pyrimidine analogs, the third kind of antifungal medication, can block pyrimidine metabolism and DNA synthesis in fungi. It possesses strong antibacterial properties against *Cryptococcus* and *Candida* and limited antibacterial properties against colored fungus and a few *Aspergillus* species. The antibacterial effect on other fungi is poor. This product is a bacteriostatic agent with a bactericidal effect at high concentrations, with greater side effects. In clinical practice, flucytosine is generally coupled with other antifungal drugs because primary or secondary resistance to this drug is common (Elgemeie et al., 2017). Echinocandin is a class IV antifungal drug that can specifically interrupt the formation of fungal cell walls without affecting human cells. Most *Candida* species, including some azole-resistant strains, respond quickly to this class of drugs. Furthermore, these drugs are fungicidal against most *Aspergillus* species, but not against *Fusarium*, *Zygobacterium*, or *Cryptococcus neoformans*. Although the fungus is less resistant to these treatments, the cost of this drug is too high for most patients to afford (Denning, 2003). Therefore, it is imperative to seek a broad-spectrum antibacterial, biosafety, and cost-effective antifungal treatment.

## Probiotics

In 2002, WHO declared probiotics as living microorganisms beneficial to the host health upon administration of sufficient doses (FAO and WHO, 2002). Pharmaceutical probiotics, food probiotics, animal probiotics for feeding, and genetically modified probiotics are several types of probiotics. Among them, drug probiotics are the most important (Venugopalan et al., 2010).

Pharmaceutical probiotics are widely used in clinical practice. Probiotics can now be used to prevent or cure clostridia-related acute diarrhea (Goldenberg et al., 2018), improve inflammatory bowel disease (IBD), irritable bowel syndrome (IBS) (Camilleri and Boeckxstaens, 2017), reduce the risk of delayed neonatal sepsis and necrotizing enterocolitis (Frost et al., 2017). Probiotics have also been reported to remove *Helicobacter pylori* (Espinoza et al., 2018), reduce the asthma incidence and severity (Arrieta et al., 2015), lessen depression (Foster and Neufeld, 2013), prevent or treat atopic dermatitis (Murch, 2001), reduce risk factors related to cardiometabolic syndrome (Rak and Rader, 2011), and even prevent cancer (Bhatt et al., 2017). Most of these applications are based on the function of probiotics that can regulate the balance of human flora and inhibit the growth of harmful bacteria. Doctors often combine probiotics with prebiotics to treat these conditions. Prebiotic is a substrate that is selectively utilized by host microorganisms, leading to the growth and multiplication of probiotics (Libertucci and Young, 2019).

Even though probiotics have been around for over a century, clinical probiotics are still understudied. Probiotics have received a lot of attention since their benefit has been recognized compared to traditional therapeutic drugs (Reid et al., 2019). Additionally, various studies have demonstrated that probiotics have inhibitory effects on pathogenic fungi such as *Candida albicans, Candida glabrata*, and *Aspergillus fumigatus* (Atanassova et al., 2003). Therefore, employing probiotics to prevent and cure fungal infections, either alone or in combination with traditional antibacterial drugs, may open up new opportunities for antifungal treatment.

There are numerous probiotics with antibacterial properties, including *Bifidobacterium, Lactobacillus rhamnosus, Sacctiaromyces boulardii*, and *Saccharomyces cerevisiae* are currently popular research subjects (Ashraf and Shah, 2014; Lam et al., 2019).

## Antifungal Ability of Probiotics

### Probiotics Can Inhibit the Filamentation of Pathogenic Fungi

Mycelium is a virulence factor found in a variety of pathogenic fungus that can stimulate fungal adherence and biofilm formation. One of the essential linkages in the pathogenic mechanism of fungal infections is the development of hyphae (Meng et al., 2019). Fungi can penetrate the epithelium and endothelium, causing tissue damage and making entry into the bloodstream in the form of hyphae. It works by releasing hydrolytic enzymes. It is also pertinent to note that invasive hyphal forms can’t be detected by the immune system. In addition, they induce a specific immune response that is mediated by macrophages (Sudbery, 2011).

Kunyeit et al. discovered the effect of *Saccharomyces cerevisiae* which derived from food as a probiotic on cell morphology and filamentation (Kunyeit et al., 2019). Significant inhibition was observed on the level of mycelial development of *Candida tropicalis and Candida parapsilosis* using the *S. cerevisiae* supernatant (10^8^ cells/mL) to treat five non-*candida albicans*. Vilela et al., also conducted experiments to induce mycelial formation in an *in vitro* environment to determine that *Lactobacillus* can inhibit the mycelial formation of *C. albicans* (Vilela et al., 2015). *C. albicans* mycelium production was also prevented in a medium supplemented with a *Lactobacillus rhamnosus* suspension (Matsubara et al., 2016). These findings suggest that the probiotic supernatant may contain an active antifungal ingredient that can prevent pathogenic fungus from filamenting, but this substance is not a protein (Guo et al., 2011).

### Probiotics Can Inhibit the Adhesion of Pathogenic Fungi

In a healthy state, a tiny amount of opportunistic pathogenic bacteria adheres to the mouth, intestines, and skin of the human body, but a small number of pathogenic bacteria do not lead to health problems. However, the overgrowth of pathogenic bacteria may cause health issues. Pathogenic bacterial adhesion is the first step in helping them invade and colonize host cells, and it also contributes to systemic infection. The inhibition of pathogenic bacterial adhesion would eventually decrease the invasion of these bacteria (Ribes et al., 2000).

To see if probiotics can prevent *C. albicans* from adhering to epithelial cells, Lohith K. et al. inoculated epithelial cells with probiotics and non-*candida albicans* under three different conditions: pre-inoculation, co-inoculation, and post-inoculation. The results showed that under pre-inoculation conditions, the adhesion inhibition rate of epithelial monolayer cells reached 95% to 99%. However, under the conditions of co-inoculation and post-inoculation, 72% to 98% inhibition of non-*Candida albicans* strain adhesion was observed (Kunyeit et al., 2019). According to *in vitro* studies, probiotics can produce antibacterial substances such as bacteriocins to regulate the microbiota (Gerding et al., 2015). Furthermore, probiotics may interact directly with lectin adhesion components to form a “physical barrier” that prevents pathogens from adhering to epithelial cells (Mukai et al., 2004).

### Probiotics Can Inhibit the Formation of Pathogenic Fungal Biofilms

Biofilm and fungal resistance are intimately connected. Biofilm is a bacterial or fungal cell colony that is attached to the surface of live or non-viable tissue and enveloped in an extracellular polymeric matrix, created by the bacterial or fungal cells themselves. It can build a diffusion barrier to prevent the penetration of antifungal drugs, protect fungal cells, diminish sensitivity and even improve resistance to antifungal drugs (Zarnowski et al., 2018). Factors associated with biofilm resistance include (i) resistance to antimicrobial agents by slowing the growth of strains; (ii) high expression of surface-induced drug resistance genes; (iii) abnormal metabolism of sterols on the membrane surface; (iv) the heterogeneity of cells resulting in the production of a large number of mycelium cells and so on (Koo et al., 2017). According to research, the sensitivity of fungi to azole drugs, amphotericin B, and other clinically commonly used antifungal drugs is significantly reduced after biofilm formation (Kowalski et al., 2020).

The development of fungal biofilms can be divided into three stages, early, middle, and mature. The bacterial cells adhere to the surface of the support in the early stages of the biofilm to form a microcolony. The microcolony then fuses and releases the matrix to form the biofilm’s base layer. Finally, the matrix is released in large quantities, accompanied by the formation of hyphae and/or pseudohyphae. The aforementioned behavior gradually complicates the biofilm structure until it evolves into a mature biofilm (Klausen et al., 2010). Fungal biofilms can mature in 48 hours in an *in vitro* environment but some substances in serum and tissue fluid might encourage biofilm formation *in vivo*, resulting in a smaller duration for biofilms maturation (Matsumoto et al., 2021). Moreover, fungal resistance emerges in the early phases of biofilm formation and grows as the biofilm matures, making deep fungal infections more difficult to cure (Eguia et al., 2020).

Probiotics have been demonstrated in numerous studies to suppress the production of fungal biofilms in the early stages. However, the inhibition of biofilms in the middle and mature phases is weak (Singhal et al., 2011; Kean and Ramage, 2019). Matsubara et al. tested the inhibitory effects of probiotic lactic acid bacteria on *C. albicans* biofilm production, using three varieties of *Lactobacilli*. The Lactobacillus culture solution had no significant effect on *Candida*’s mature biofilm, but it inhibited biofilm formation in the early stages of *Candida* (Matsubara et al., 2016). The *in vitro* experiment of Smith et al. yielded similar results. *C. albicans* biofilm formation in the *Lactobacillus acidophilus* culture solution is lower than the control group that had sterile semi-skimmed milk instead of probiotic culture solution (Smith et al., 2012).

In addition, Nover and Huffnagle (2004) investigated the effects of probiotic live cultures, culture supernatants, and dead cultures on the morphogenesis of *C. albicans*. They discovered that the supernatant derived from a 2-hour probiotic culture suppressed *C. albicans* embryonic tube development. On the other hand, the supernatant derived from a 24 hrs probiotic culture fully suppressed *C. albicans* budding. This finding suggests that the soluble antibacterial substance in the culture supernatant builds up over time, resulting in a disparity in inhibition. This indicates that probiotics have the fastest fungal inhibition time.

### Mechanism of the Probiotics for Pathogenic Fungal Inhibition

Many *in vitro* inhibitory studies with pathogens have demonstrated that probiotics can suppress fungi, but the mechanisms by which probiotics protect against infection remains a mystery (Hasslöf et al., 2010; Coman et al., 2014; Ujaoney et al., 2014). Different hypotheses have been proposed for their antifungal activity. The ability of a probiotic to inhibit pathogenic fungi is most likely influenced by interactions between pathogenic fungi and probiotics (Matsubara et al., 2016). When probiotics are co-cultured with a pathogen, they can compete with the pathogen for receptor sites/binding sites, nutrients, and growth factors (Amara and Shibl, 2015; Kunyeit et al., 2019; Lam et al., 2019). Furthermore, the production of hydrogen peroxide (H_2_O_2_) and the release of organic acids, such as lactic and acetic acid are also helpful in the antifungal mechanisms of these probiotics (Ashraf and Shah, 2014; Bhatt et al., 2017). More molecular and biological research, particularly employing gene expression and associated technology is needed to confirm the antifungal mechanism of probiotics.

## Future Prospects

We urgently need to discover antifungal compounds with new mechanisms of action to deal with the rising number of cases of fungal infections. Furthermore, better management is required to limit the resistance of pathogenic bacteria to existing antifungal drugs and to develop novel disease control strategies to avoid over-reliance on drug treatment.

Although the antibacterial mechanism of probiotics is not yet clear, it is evident that they have a strong antifungal effect. Therefore, probiotics have enormous development potential as a safe and cost-effective new method of treating fungal infections. Furthermore, while live probiotic cells and their metabolites can have synergistic effects, probiotics cell-free secretions also have an antibacterial effect, extending the application spectrum of probiotics. Therefore, exploring the antibacterial mechanism of probiotics and discovering more antibacterial probiotics that can be used in clinical treatment may become a focus of probiotic research in the future.

## Author Contributions

YY, FG, and CW conceived and designed the study. JW and SH collated and collected data. JW, SH, and YY drafted the manuscript. All authors contributed to the article and approved the submitted version.

## Funding

Ministry of Science and Technology of the People’s Republic of China (grants 2018ZX10101003, 2018ZX10712001).

## Conflict of Interest

The authors declare that the research was conducted in the absence of any commercial or financial relationships that could be construed as a potential conflict of interest.

## Publisher’s Note

All claims expressed in this article are solely those of the authors and do not necessarily represent those of their affiliated organizations, or those of the publisher, the editors and the reviewers. Any product that may be evaluated in this article, or claim that may be made by its manufacturer, is not guaranteed or endorsed by the publisher.
